# Perioperative pathways for children with neurodevelopmental conditions and behaviours that challenge: An evaluation of parent experiences for service improvement

**DOI:** 10.1177/17504589241253487

**Published:** 2024-08-06

**Authors:** Natalie Ong, Hema Ahuja, Jonathan de Lima, Gail Tomsic, Pankaj Garg, Natalie Silove, Bobbi Henao Urrego, Andrew Weatherall

**Affiliations:** 1 School of Public Health, Faculty of Medicine and Health, The University of Sydney, Camperdown, NSW, Australia; 2 Child Development Unit, The Children’s Hospital at Westmead, The Sydney Children’s Hospitals Network, Westmead, NSW, Australia; 3 Department of Anaesthesia, The Children’s Hospital at Westmead, The Sydney Children’s Hospitals Network, Westmead, NSW, Australia; 4 School of Women’s and Children’s Health, University of New South Wales, Sydney, NSW, Australia; 5 Specialist Intellectual Disability Health Team, Department of Community Paediatrics, South Western Sydney Local Health District, Warwick Farm, NSW, Australia; 6 Executive Unit, The Sydney Children’s Hospitals Network, Westmead, Australia; 7 Division of Child and Adolescent Health, The University of Sydney, Camperdown, NSW, Australia

**Keywords:** Adapted care pathway, Quality and safety, Qualitative research, Neurodevelopmental conditions, Autism spectrum disorder, Developmental disability

## Abstract

Previous research shows that children and young people with neurodevelopmental conditions experience greater challenges accessing care in hospitals and having their health needs met.

**Aim:**

To elicit experiences of parents of children with neurodevelopmental conditions using a new perioperative pathway.

**Method:**

Parents of children accessing an adapted perioperative clinical pathway in a tertiary children’s hospital between July 2019 and December 2020 were invited to participate. A mixed method study was conducted comprising a short survey questionnaire followed by telephonic interviews.

**Results:**

From 67 postal surveys sent out, 20 were completed. Six out of 20 parents participated in phone interviews and one parent submitted written prose. Parents were positive about their experiences. Six themes emerged: *Negative past experiences* (highlighting the need for adapted perioperative pathways); *Reasonable adjustments* (improving child and parent’s hospital journey); *Facilitating communication, convenience and collaboration; Parent’s satisfaction and relief; Barriers to overcome and Areas in need of improvement* were discussed.

**Conclusion:**

Parents of children with neurodevelopmental conditions report great satisfaction and relief from their experiences of a more efficient, streamlined and stress-free way for their child to have tests or procedures done. Parents report improved communication, convenience and collaboration with staff resulted in timely, safe and high-quality care.

## Introduction

Children or young people with neurodevelopmental conditions are known to have complex, special health care requirements compared to the general population due to comorbidities associated with neurodevelopmental diagnoses. Hospital visits can be stressful when scheduled for therapeutic and diagnostic procedures, leading to anxiety and behavioural escalation in an unfamiliar environment. Negative past experiences can trigger anxiety and trauma for the child, their family and staff when presenting to such a setting (Jensen et al 2020, Nelson & Amplo 2009, Schieve et al 2012). Appointments may be missed and care delayed (Bultas 2012, Liddle et al 2018) because of the child or young person fearing a return to hospital. In addition, anxious staff may be reluctant to provide care (Jensen et al 2020, Johnson & Rodriguez 2013, Liddle et al 2018, Pisula & Porębowicz-Dörsmann 2017). Health care practitioners are required to manage children with behaviours that challenge but feel unprepared to handle such behaviours (Johnson & Rodriguez 2013, Tufford & Newman 2012). The effect can impact the safety and wellbeing of the child, staff coping mechanisms and fitness to work (Gimbler Berglund et al 2016, Graham et al 2009, Mimmo et al 2019).

Appropriate paediatric sedation and anaesthesia are considered a standard of care for children undergoing invasive diagnostic and therapeutic procedures. While standard paediatric planning processes and care are sufficient for typically developing children, there is a need for additional considerations for those neurodiverse children in health care environments (Benich et al 2018, Lindberg et al 2012). There is a paucity of research exploring the influence of specialised adaptive care plans (ACPs) on the unique needs of children with developmental disabilities in perioperative hospital settings (Kennedy et al 2016, Liddle et al 2018, Lindberg et al 2012).

The Quiet pathway (Sydney, Australia) is a dedicated perioperative pathway provided by the Department of Anaesthesia at (The Children’s Hospital at Westmead), a major paediatric teaching hospital in (Sydney, Australia). It is designed to provide an adapted care pathway to facilitate successful procedural outcomes and improve patient experience, safety and quality of care for children and young people with neurodevelopmental conditions who have had difficulties accessing standard care.

Patients are identified by health practitioners and pre-anaesthetic clinic coordinators, during bookings, planning processes or direct disclosure by families. An electronic request for admission is completed by the referring health practitioner, identifying the need for the pathway. The pre-anaesthetic clinic coordinator is informed by email of referred patients.

After the initial notification, the triage anaesthetist reviews the child’s available documentation regarding previous health encounters with clinicians and clinical services involved in the patient’s care. Subsequently, the anaesthetist consults the referring clinician and the child’s parent to obtain relevant information about the child: pre-existing conditions; key behavioural issues either impacting or triggered by the interaction; information about non-pharmacological approaches that are supportive for the patient and previous experiences with sedatives or behaviour-modifying medications.

A vital component of perioperative care is flexibility in processes. Minimising fasting time, starting pre-medication prior to admission and arranging admission close to the procedure or via streamlined pathways can facilitate an optimal waiting environment (Kersten et al 2023, Ong et al 2022). Before admission, the triage anaesthetist provides a brief summary and notification to the procedural anaesthetist and other staff members who are to be involved in the child’s care such as the Day Stay Unit, anaesthesia or theatre staff and preadmission clinic staff. If a child is susceptible to distress in a hospital environment, expedited discharge from the recovery ward is considered with a plan to be followed up in the community by the nursing staff or paediatrician and by phone call from the anaesthesia department post discharge.

### Aims of the study

This pilot study aims to elicit the experiences of parents of children with neurodevelopmental conditions through their participation in the adaptive perioperative pathway to identify barriers and facilitators to improve the ongoing development of this service.

## Methods

### Setting and recruitment of participants

This study was conducted in the Anaesthesia Department at the (Sydney, Australia). The study’s participants were parents of children who had accessed the perioperative pathway between July 2019 and December 2020, over a period of 18 months.

### Inclusion criteria

1. Parents of children and young people (aged 0–16 years) with neurodevelopmental conditions for whom overstimulation and sensory overload tend to trigger fear, aggression or self-harming behaviours.
2. Parents of children with significant needle and mask phobias or mental health disorders in whom anxiety and behaviours make routine health care extremely difficult.

### Exclusion criteria

1. Parents of children who accessed the pathway outside the 18-month period.
2. Parents of children who did not meet inclusion criteria.

### Study design

This is a mixed method research study consisting of a survey and semi-structured interviews. Participation in the study was voluntary and participants were allowed to withdraw at any stage of the project. Mixed methods was chosen due to its comprehensive nature of providing a combination of quantitative and qualitative data to enrich our understanding of the parent experience (Tariq & Woodman 2013).

This study was approved by the (Sydney Children’s Hospital Network Human Research Ethics Committee).

## Methods

### Phase 1 (survey)

Surveys were mailed in February 2021 to 67 parents of the children who had accessed the perioperative pathway between July 2019 and December 2020. After two weeks, a follow-up phone call was made to the potential participants who had not returned the survey. The surveys were re-sent in April 2021 to parents reporting non-receipt. The survey was designed as a short ten-minute questionnaire, comprising 20 questions, a combination of questions being scored on a 5-point Likert-type scale and free-text responses. Parent demographic information and details of their child’s anaesthetic procedure were accessed from the Anaesthesia Department medical records. As a prompt, details of their child’s specific admission were provided on each individual survey to help the parent recall the particular perioperative admission for their child.

For more information, please see Supplemental Material 1.

Each parent was deidentified by assigning a unique study code number with the identifying list only known to the primary investigator. Participating parents who completed the survey returned it by post through a reply-paid envelope. This was collated by the department secretary and forwarded to the primary investigator.

### Phase 2 (phone interviews)

Parents who consented for follow-up interviews in the survey were contacted by the primary investigator. The purpose of these telephonic interviews was to engage in a more in-depth description of the experience of the adapted pathway. The phone interviews were audio-recorded and transcribed by an independent transcription service and cross-checked with the primary investigator’s handwritten notes. Any identifying information was removed from the transcripts prior to data analysis.

The interview brief is included in Supplemental Material 2.

## Data analysis

### Survey data

The data from the survey forms were coded electronically and analysed using MS Excel. Demographic information and key aspects of the hospitalisation experience were extracted, for example preadmission, admission, overall experience, hospital staff interaction with the children and previous health experiences. This showed that parent and child experience had improved with the introduction of the new pathway. We then went on to interview the families in more depth to better explore their reasons for change in care.

### Interview data

The interviews were initially evaluated and coded separately by the primary investigator and the co-investigator. The primary investigator is a paediatric trainee with experience in general and developmental paediatrics. The co-investigator is a developmental paediatrician with experience in intellectual or developmental disability and mixed methods research. Investigator bias was addressed through a bracketing exercise where the investigators independently and reflexively took notes on how their interactions with patients and families in their clinical roles would influence their interpretation of the data. Investigator reflections were discussed highlighting any issues and ways to mitigate any bias (Tufford & Newman 2012). These codes were discussed for consensus and thematic analyses derived from these discussions with the research team. The qualitative analysis of the interviews was conducted by using an abductive approach using the Framework Method (Bultas 2012, Gale et al 2013).

The Framework Method involves organising data into codes and categories. This method helps to identify similarities and differences in qualitative data, and subsequently focuses on association between different parts of data, looking for outcomes in the form of central identified themes. This framework structures and generates succinct data optimising development of themes from service feedback data.

### Community involvement

This project was based on feedback from parents of children with autism though they were not actively involved in the design, implementation or evaluation of the study.

## Results

### Survey results

A total of 67 surveys were posted to the potential participants (parents of children who were triaged through the perioperative pathway from July 2019 to December 2020). Twenty parents completed and returned the surveys to the Anaesthesia Department. Six out of 20 consented to participate in phone interviews, and one parent shared a letter illustrating their experience with the perioperative pathway. Please see Tables 1 and 2 for demographic information on parent and child.

**Table 1:** Demographic Information: parent participants

Participant or parent	Participant or parent status	Parent age category (years)	Educational status	Occupation
1	Single	51–60	Secondary school	Not working
2	Dual	30–40	Tafe or diploma	Not working
3	Dual	51–60	University degree	Employed
4	Dual	41–50	Secondary school	Employed
5	Dual	30–40	Tafe or diploma	Not working
6	Dual	30–40	Secondary school	Not working
7	Foster parent	51–60	Master’s degree or postgraduate	Employed
8	Dual	30–40	Tafe or diploma	Not working
9	Dual	51–60	Master’s degree or postgraduate	Employed
10	Dual	41–50	Master’s degree or postgraduate	Employed
11	Dual	30–40	University degree	Not working
12	Dual	30–40	Tafe or diploma	Not working
13	Dual	30–40	Tafe or diploma	Not working
14	Single	41–50	Tafe or diploma	Not working
15	Dual	41–50	Secondary school	Not working
16	Dual	51–60	Tafe or diploma	Not working
17	Dual	41–50	Secondary school	Employed
18	Dual	30–40	University degree	Employed
19	Dual	41–50	Master’s degree or postgraduate	Employed
20	Single	30–40	Tafe or diploma	Employed

Source: Author.

**Table 2:** Demographic information: child participant

		Total number of children
Age	<10 years	7
	10–15 years	8
	>15 years	5
Sex	Male	12
	Female	8
Main diagnosis	Autism	18
	Non-autism	2
Reasons for triage under perioperative pathway	Intellectual disability and behaviours that challenge	1
	Attention-deficit hyperactivity disorder, oppositional defiant disorder and behaviours that challenge with anxiety (needle phobia)	1
	Autism and behaviours that challenge with anxiety	18

Source: Author.

In our cohort of children, the most common interventions performed under anaesthesia were imaging alone or with intervention, followed by other miscellaneous surgical procedures. For more information, please see Tables 3 and 4.

**Table 3:** Procedures conducted under anaesthesia for children who were triaged through perioperative pathway

Procedure under anaesthesia	Frequency of procedure N = 20 (no children)
Imaging (MRI)	4**
Imaging with intervention	
Angiography and embolization	5*
Dental	4**
Insertion of intrauterine device	1
Removal of plates	1
Heller’s myotomy and fundoplication	1
MRCP	1
Tonsillectomy and adenoidectomy	1
Eye and ENT examination	1
Examination of Pilomatrixoma	1

** one child presenting for MRI and dental.

* represents one child undergoicng repeated procedure.

MRCP: magnetic resonance cholangiopancreatogram.

**Table 4:** Most important aspects of preadmission preparation cited by parents

• Discussion and provision of a plan prior to the procedure day • Contact from staff to gather information and update them on what to expect on the day • Information regarding parking • Directions where to present with their child

MRI: magnetic resonance imaging; ENT: ear, nose and throat.

**Figure 1:**
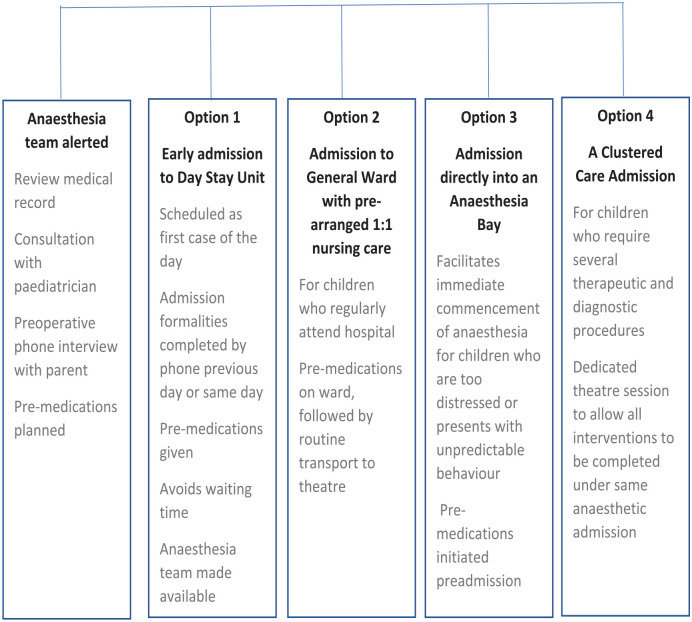
Perioperative pathway – the five care plan

**Figure 2:**
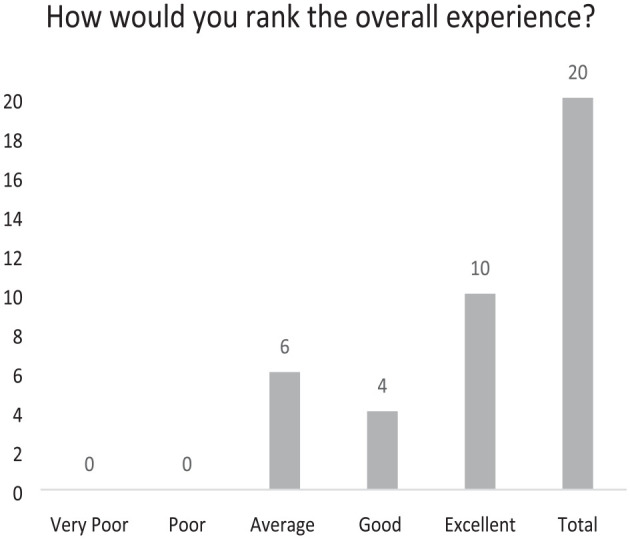
Overall experience

The results from the survey are described in the tables (see Figures 1–5).

### The Five Care Plan

### Overall Experience

### Support provided during admission

**Figure 3:**
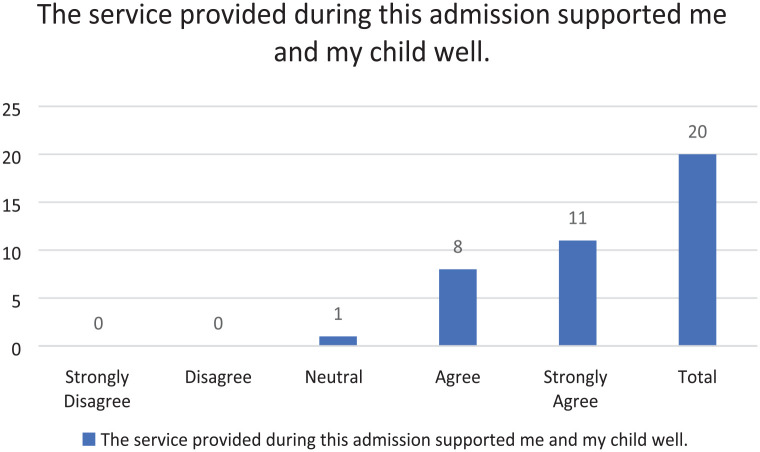
Support provided during admission

### Previous experience during other hospital admissions

**Figure 4:**
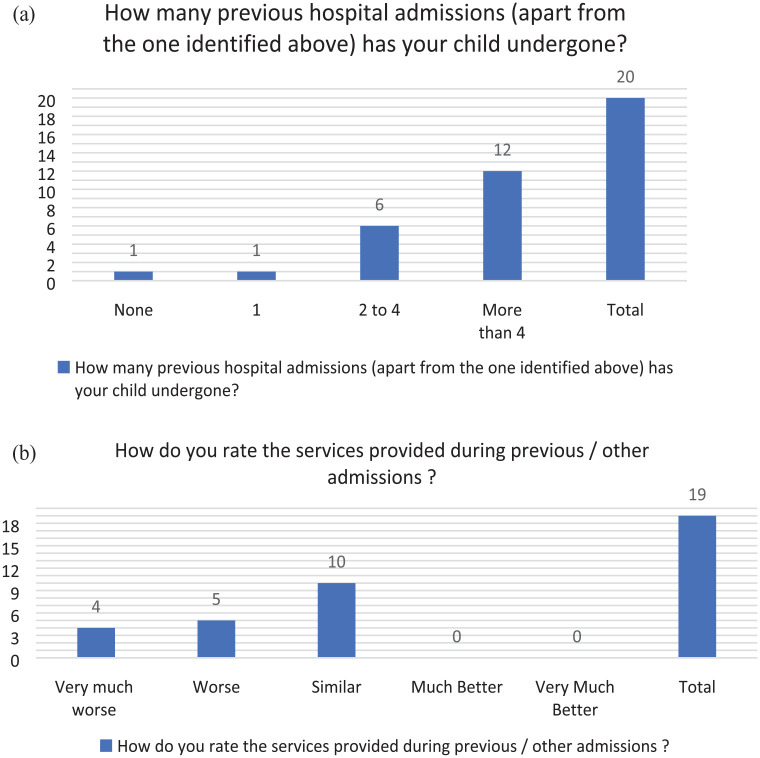
(a) Previous experience during other hospital admissions. (b) Rating of previous hospital admission experience

### Staff interaction

**Figure 5:**
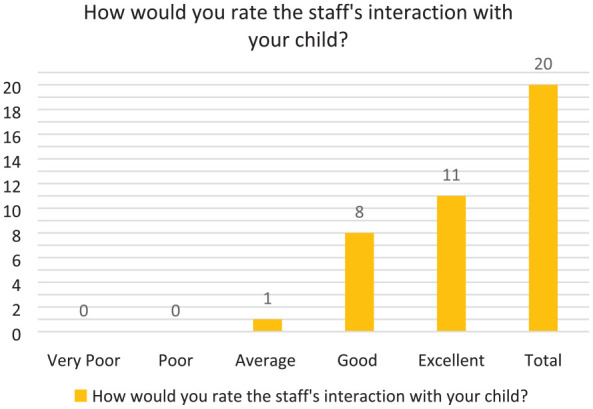
Staff interaction

### Interview results

The Framework methodology was used to conduct a thematic analysis of the interview transcripts. Six themes emerged from the synthesis: *Negative past experiences highlight the critical need for adapted perioperative pathways; Reasonable adjustments improve the child and parent’s hospital journey; Things that helped: communication, convenience and collaboration; Parent’s satisfaction and relief for a more positive experience; Barriers to overcome and Areas to improve.*

### Negative past experiences highlight the critical need for adapted perioperative pathways

Some parents shared their previous hospital experiences, primarily involving lack of planning and the inclusion of special provisions to support a successful admission for children with intellectual or developmental disabilities or behaviours that challenge.

One parent shared previous hospital experience in which their child’s behaviour deteriorated due to delayed preparation and administration of premeds. Perioperative preparations could have avoided the situation according to the parent, underscoring their concern about the current admission.

*Just, previously, it was just very challenging like, when I was saying when you come in and you’re trying to sign paperwork but your child is going absolutely crazy. Because sensory wise it’s just all too much for him. And then, like when we went through if you’re waiting sort of a long time for those pre op meds, he would be very agitated and you did start sort of, because he’s hungry as well from fasting. He would start going through everyone’s bag*. (Participant 14: p23)

Another parent reported similarly where lack of timely administration of premeds led to the child’s behavioural escalation leading to the need for restraint.

*As I said that the nursing staff, even though they’re wonderful didn’t anticipate, . . . we were warning them that the waiting time wasn’t going to end very well. He was still made to wait, . . . they still want me to fill out all the forms, prior . . . going in to have the procedure. Also, we had given him some valium beforehand, but he was too escalated for it to take effect . . . and he became quite distraught and violent.* (Participant 16: p27)

Some parents shared previous negative experiences from lack of planning resulting in prolonged waits and fasting times causing agitation in their child.

*It’s hard to manage them being hungry but it’s also hard to manage a 14-year-old who can’t understand why he’s not allowed to eat.* (Participant 19: p29)

*With that experience fresh in our minds, we experienced trepidation and anxiety in the lead up to the visit for the MRI.* (Participant 3: p3)

One parent reported a lack of adequate information transfer between different hospital teams, resulting in the postponement of a long-awaited procedure.

*I can remember something we’ve been booked in for the scan and he was nil by mouth and we were waiting and waiting and it got to two o’clock, and the theatre staff has said sorry. The MRI receptionist had said, we can’t do him today because we haven’t got clearance that he doesn’t have plate, metal in his head, clips. I said, but he doesn’t have clips and she said yes, but we don’t have a letter from treating hospital. We’ve waited 10 months for this procedure.* (Participant 19: p37)

Another parent thought the hospital environment was unsuitable for her child reporting noise crowding and a long waiting time negatively impacting the child’s cooperation. A less than ideal compromise on managing at home had to be made.

*You just can’t make a child like that, do what they don’t want to do. So, yeah, it helped that we have managed him from home and we focused on the one big thing that he had to do rather than all the other things he had to be subjected to.* (Participant 10: p43)

### Reasonable adjustments improve the child and parent’s hospital journey

The most consistent theme was that parents appreciated the preadmission preparation.

Premeds were successfully administered in some cases enabling the child to be transferred to theatres with little resistance. As their child moved from one place to another, none of the parents experienced long wait times. The plan included having the correct number of staff who knew the child and collaborated with the parent to ensure care was delivered efficiently, and a clear postoperative plan was provided at time of discharge.

*Everyone seemed to know who he was and what the issues were before we got there, which was awesome. So, I didn’t have to explain it, in front of him.* (Participant 9: p36)

*So, it’s a much better streamlined process, and less traumatic for him and for us, and it wasn’t only preoperatively, it was postoperatively as well.* (Participant 9: p25)

*The anesthetists came and seen him the night before and got a good history on what he’s like and then, that made everything, the next day run a lot smoother. Because they were expecting him and they knew his behavior, they knew his autism, you know, it sort of sped the process along quite a bit.* (Participant 14: p7)

### Things that helped: Communication, convenience and collaboration

Parents felt perioperative communication with staff addressed their concerns. They felt supported and valued being included in the planning discussion for their child’s procedure.

Parents appreciated the perioperative pathway arrangement making it extremely convenient scheduling all of their child’s appointments and procedures under a single anaesthesia administration in collaboration with the multiple teams.

*I think what was really key to the whole success of the day was the fact that all the teams were available at the same time for the procedure. So, it was kind of very opportunistic, to get that. So he needed a few things looked at; so he needed his teeth and his eyes and his ENT looked at and all of that was done at the same time which is great. That saves him having a series of general anaesthetics and a series of admissions and it also means that we’ve got a better collaboration between the teams.* (Participant 19: p21) *. . .* and (change)

*So, I think that communication between the anaesthetic department and the parent is a very good approach.* (Participant 2: p16)

*So we went straight into a room where there was like the chair bed kind of thing, and then he went from there pretty much straight away, so he was not lingering around there waiting around and then straight into the room into the operation.* (Participant 9: p27)

### Parent’s satisfaction and relief for a more positive experience

Parents appreciated the teamwork and professionalism exhibited by staff noting excellent interaction and communication with their child, and their child being kept comfortable while receiving postoperative care. All parents expressed full support and confidence in the perioperative pathway services.

*It’s because he is light sensitive, it’s a very bright atmosphere and he does find that quite difficult, so he’ll quite often want the blankets, all over his head . . . and I found this time, I was in there, the nurses were very understanding of that. Like previously there was sort of like, no J, we need to have the blankets down, we need to watch you we need to observe you. But this sort of like, this time around they were a lot more understanding.* (Participant 14: p129) *. . .* and

*On the day itself, it would not have been possible to have received better medical treatment or care or to have had a better outcome. Everything went as planned without any complications and that is due entirely to the hospital staff. I simply cannot commend them highly enough we are very grateful that they removed so much of the stress from the whole experience.* (Participant 2: p8)

*Absolutely. I wish all the hospitals have had this sort of facility.* (Participant 16: p25)

### Barriers to overcome

Although the parents acknowledge the added efforts of staff supporting their child or young person, a small number of parents reported ongoing glitches with perioperative services. This included staff rushing interactions with their child, pressured by time constraints. Parents felt better management was needed to help their child to feel more comfortable and at ease.

*We have had problems, a few times with the way she was spoken to. And there was one time that she was told to hurry up to take the mask because the doctor had an emergency to attend to. So, there has been situations like that. But overall, I can’t, I really can’t fault them.* (Participant 2: p21)

### Areas to improve

Although the perioperative pathway was widely supported and appreciated by participating parents, some recommendations for improvement were made. These included addressing noise in the main waiting area to allay anxiety or behavioural escalation in children sensitive to noise or crowds.

*It’s a very noisy area with the children coming out from their procedures, and you’re sitting there with a child who’s going into a procedure, who has sensory issues, and the bright lights and their noises can usually keep them still quite anxious, even though they’re on Lorazepam.* (Participant 2: p10)

The parents stated preference for a separate, quiet waiting area to avoid potential stress and anxiety from being in a crowded place like a hospital.

*Yes. I think the only thing is the discharge procedure, if there can be a quiet pathway for discharge as well. I think that could be. I don’t know how hard that would be to organize, probably near impossible with the hospital system these days, but I think the quiet pathway in and out would be fabulous.* (Participant 16: p57)

Some parents emphasised the importance of having a disability liaison officer or nurse to assist communication between family and the teams to facilitate smooth hospital encounters.

*What makes the whole process about coming to hospital easier is having a good relationship and a communicated relationship with the senior nurse specialist, representing the department that you’re dealing with. Not only can they liaise with you and the doctor any concerns prior to admission. They can help set up any follow up appointments and if we are coming down for a scheduled appointment and we have other things going on, they help liaise things, to the best of their ability, so it’s not overwhelming for the child.* (Participant 2: p70)

*But certainly, some kind of hospital community interface or some kind of, whether it’s a liaison officer or Disability Liaison Officer, that can coordinate with the team would be incredible.* (Participant 19: p31)

## Discussion

Challenging health care encounters for children with neurodevelopmental conditions are experienced universally (Mimmo et al 2018, Ong et al 2017, Oulton et al 2020). Behaviours that challenge often pose a significant impediment for clinicians to perform necessary procedures due to their lack of experience and training in this area (Carter et al 2016). There are emerging studies internationally promoting improved hospital services for children with disabilities (Gimbler Berglund et al 2016, Lindberg et al 2012, Mimmo et al 2019, Oulton et al 2018). It has been shown that these barriers can be overcome by services set up to support individualised care pathways (Garrick et al 2022, Liddle et al 2018, Litwin & Sellen 2023, Viotti et al 2020, Wood et al 2019). Clark et al described an ACP to help children with developmental disabilities navigate a healthcare experience (Clark et al 2019). Similarly, Balakas et al (2015) advocate using a nurse-initiated ACP tool to improve the perioperative care of children and adolescents with challenging behaviours (Balakas et al, 2015). These programmes serve not only to improve patient experience and reduce harm but also improve clinical efficiencies, cost effectiveness and potentially prevent staff burnout.

The perioperative pathway services for children with neurodevelopmental conditions is an example of an adapted care pathway offered by the anaesthesia department at a tertiary children’s hospital, developed to enable efficacious access to services for children with intellectual and developmental disability and behaviours that challenge. This initiative was to address the gaps between the need and the scarcity of locally available individualised perioperative services. This study provides insight into the experiences of parents who received these services for their child, often previously challenged by access barriers and staff workforce not equipped to meet their needs.

In this study, most of these children have had multiple past admissions to different hospitals that have led to fears anticipating their current admission. We found the majority of parents reported positive experiences and improved care, strongly recommending the continuation of this service. The findings revealed an explicit need for an individualised care pathway for such children.

## Limitations of the study

We acknowledge the lack of representation from Aboriginal and Torres Strait Islander, non-English-speaking, new arrival and out-of-home care groups. However, benefits from the perioperative experience pathway may also benefit vulnerable groups as well warranting further research in this area.

Corresponding health care staff were not interviewed to obtain feedback on their experiences and how the services could be improved in this iteration. Future evaluations will include staff, patient and stakeholder perspectives to improve triangulation of the data obtained.

Recall time of the event encountered was potentially lengthy. It may have contributed to the limited response from parents.

The relatively low response participation rates are acknowledged. However, previous research has highlighted and reflected the busyness of such parents caring for children with complex disabilities (Cantero-Garlito et al 2020, Ngo et al 2012). They have to juggle multiple appointments, caring for the child, the family, work and managing home life.

The study period occurred within the phases of COVID restrictions. This may have added to the challenges of parents responding to the survey in a timely manner.

## Conclusion

Parents of children with neurodevelopmental conditions report great satisfaction and relief from their experiences of a more efficient, streamlined and efficient way for their child to have tests or procedures done in a stress-free way as possible. Parents found that improving communication, convenience and greater collaboration between themselves and staff made for a highly effective adaptive care pathway that allows children with neurodevelopmental conditions and behaviours that challenge to receive customised care, reduce multiple anaesthetic procedures, improve the patient experience and provide safe and high-quality care. The potential for such initiatives to be translated into other settings, or included in staff training and systems improvement strategies, should be further explored in future research.

## Supplemental Material

sj-docx-1-ppj-10.1177_17504589241253487 – Supplemental material for Perioperative pathways for children with neurodevelopmental conditions and behaviours that challenge: An evaluation of parent experiences for service improvement Supplemental material, sj-docx-1-ppj-10.1177_17504589241253487 for Perioperative pathways for children with neurodevelopmental conditions and behaviours that challenge: An evaluation of parent experiences for service improvement by Natalie Ong, Hema Ahuja, Jonathan de Lima, Gail Tomsic, Pankaj Garg, Natalie Silove, Bobbi Henao Urrego and Andrew Weatherall in Journal of Perioperative Practice

sj-docx-2-ppj-10.1177_17504589241253487 – Supplemental material for Perioperative pathways for children with neurodevelopmental conditions and behaviours that challenge: An evaluation of parent experiences for service improvement Supplemental material, sj-docx-2-ppj-10.1177_17504589241253487 for Perioperative pathways for children with neurodevelopmental conditions and behaviours that challenge: An evaluation of parent experiences for service improvement by Natalie Ong, Hema Ahuja, Jonathan de Lima, Gail Tomsic, Pankaj Garg, Natalie Silove, Bobbi Henao Urrego and Andrew Weatherall in Journal of Perioperative Practice

sj-docx-3-ppj-10.1177_17504589241253487 – Supplemental material for Perioperative pathways for children with neurodevelopmental conditions and behaviours that challenge: An evaluation of parent experiences for service improvement sj-docx-3-ppj-10.1177_17504589241253487 for Perioperative pathways for children with neurodevelopmental conditions and behaviours that challenge: An evaluation of parent experiences for service improvement by Natalie Ong, Hema Ahuja, Jonathan de Lima, Gail Tomsic, Pankaj Garg, Natalie Silove, Bobbi Henao Urrego and Andrew Weatherall in Journal of Perioperative Practice This article is distributed under the terms of the Creative Commons Attribution 4.0 License ( https://creativecommons.org/licenses/by/4.0/) which permits any use, reproduction and distribution of the work without further permission provided the original work is attributed as specified on the SAGE and Open Access pages ( https://us.sagepub.com/en-us/nam/open-access-at-sage).
