# Supplementary material for: Perioperative pathways for children with neurodevelopmental conditions and behaviours that challenge: An evaluation of parent experiences for service improvement
Source: J Perioper Pract. 2024 Aug 6;35(6):258–68. doi: 10.1177/17504589241253487 (PMC12092945; doi:10.1177/17504589241253487)
Supplement: sj-docx-1-ppj-10.1177_17504589241253487 – Supplemental material for Perioperative pathways for children with neurodevelopmental conditions and behaviours that challenge: An evaluation of parent experiences for service improvement [file sj-docx-1-ppj-10.1177_17504589241253487.docx]

**Carers Interview Script**

***The following questions will be used to gather information by phone interviews (Including above not exclusive of other information provided by parents)***

*Hello, my name is Dr [] from []. We have received your consent to participate in a phone interview which is a part of our research project “Perioperative Pathways for Challenging Kids--Experiences of Carers of Children”. Thank you for your time and participation in this research project.*

*Is this a good time for a phone interview?*

*If yes,*

*Would you like to continue in English, or would you like to use a phone interpreter?*

*This call be will be recorded for data analysis purpose and I will also be taking written notes. Any information you provide to us will be confidential and will not affect how your child will be managed in the future at the [].*

*Do you have any questions regarding the research? Let me reassure you, you and your child’s identifying information will remain confidential. You can also withdraw anytime. Are you happy to proceed?*

1. *Recently your child attended the children's hospital to have a procedure done under anaesthetic. Can you tell me about your experience with it?*

| *Description of the procedure and why it was needed*  *Information provided; information gathered from parent- child’s main issues were addressed by the team?*  *Preparation for the day – parent initiated, or staff initiated*  *Carer/Parents main concerns regarding their child’s procedure and why? What were they anticipating?*  *What was the child’s diagnosis?*  *Description of blow by blow account of what happened, how did they feel it went, etc. What was on their mind?*  *Medical staff attitudes and interaction with your child, hospital environment eg parking, access, roles and responsibilities, communication, smoothness of process, post-discharge care and follow up* |
| --- |

1. *Has your child ever had a previous surgical procedure under anaesthesia, whether be it at the [] or elsewhere? If yes*

*Can you me about that experience?*

*3) How does your previous experience compare with the current one?*

*4)Can you tell me about your experience in living with and provision of care for*

*your child and how this impact your capacity to access services.*

| *Finding out the diagnosis and how it impacted their family functioning*  *Accepting the diagnosis*  *Learning to care for their child and address their needs*  *Everyday difficulties /struggle of having a child with ID-DD and challenging behaviours*  *Impact on parents/ carer’s wellbeing (physical health, psychological/ emotional/ relational)*  *Impact on family life and siblings*  *Supports received – formal and informal eg. community/ health services/ agencies* |
| --- |
