# Supplementary material for: Perioperative pathways for children with neurodevelopmental conditions and behaviours that challenge: An evaluation of parent experiences for service improvement
Source: J Perioper Pract. 2024 Aug 6;35(6):258–68. doi: 10.1177/17504589241253487 (PMC12092945; doi:10.1177/17504589241253487)
Supplement: sj-docx-2-ppj-10.1177_17504589241253487 – Supplemental material for Perioperative pathways for children with neurodevelopmental conditions and behaviours that challenge: An evaluation of parent experiences for service improvement [file sj-docx-2-ppj-10.1177_17504589241253487.docx]

***Perioperative Pathway for Challenging Kids- Experiences of Carers of Children***

***A Survey***

| **Your child underwent anaesthesia at the Children’s Hospital at Westmead on: (***date)*  **For*…………………………………………………………………………………………………………………………………………*** |
| --- |

**About your experience during this admission**

**Please rate your experience at the children’s hospital Westmead**

|  | **Very Poor** | **Poor** | **Average** | **Good** | **Excellent** |
| --- | --- | --- | --- | --- | --- |
| **How would you rank the pre-admission experience?** |  |  |  |  |  |
| **How would you rank the admission experience?** |  |  |  |  |  |
| **How would you rank the overall experience?** |  |  |  |  |  |
| **How would you rate the staff's interaction with your child?** |  |  |  |  |  |

|  | **Strongly Disagree** | **Disagree** | **Neutral** | **Agree** | **Strongly Agree** |
| --- | --- | --- | --- | --- | --- |
| **The service provided during this admission supported me and my child well.** |  |  |  |  |  |

**Please describe what aspect of the service worked well.**

____________________________________________________________________________________

____________________________________________________________________________________

**Please describe what aspect of the service did not work well.**

**____________________________________________________________________________________**

**____________________________________________________________________________________**

**About previous experiences during hospital admissions**

**We are interested to hear about any previous experiences you have had accessing health services.**

**How many previous hospital admissions (apart from the one identified above) has your child undergone?**

None  1  2-4  more than 4

**If you have admitted your child to the hospital at any other time, please kindly comment on your and your child’s experience.**

___________________________________________________________________________________

___________________________________________________________________________________

|  | **Very much worse** | **Worse** | **Similar** | **Much Better** | **Very Much Better** |
| --- | --- | --- | --- | --- | --- |
| **How do you rate the service provided during previous/other admissions?** |  |  |  |  |  |

**About you- the primary carer**

**I am a….**

Single parent  Dual parents  Foster carer  Legal Guardian  A relative

Other……………………….

**Your age:**  <30  30 – 40  40-50  50 -60  >60

**Carer Education:**

Primary School  Secondary School  TAFE/ Diploma

University Degree  Master’s Degree /Post Graduate Qualification

**Carer’s Occupation:**

Employed  Self-employed  Retired  Not working

**Language:**

English speaking background  Non-English-speaking background

(please specify) _____________________________________

**About the challenges you face**

**In providing healthcare for your child, what are the three (3) most significant challenges you face? (Rank from highest to lowest). You may provide details if you want.**

| **Rank (number only three)** |  | **Comment** |
| --- | --- | --- |
|  | Transport |  |
|  | Physical safety |  |
|  | Workplace concerns |  |
|  | Financial Constraints |  |
|  | Social isolation |  |
|  | Balancing the needs of other children and family members |  |
|  | Family life balance |  |
|  | Managing appointments and multiple commitments |  |
|  | Accessing / arranging/ managing support services |  |
|  | Carer’s physical health |  |
|  | Carer’s mental health |  |
|  | Other |  |

**How do you rate your financial security?**

Extremely vulnerable  Vulnerable  Stable  Secure

**About the support you receive**

**What support do you currently receive?**

Actively engaged GP  Public Health Service  Private Health Provider

Specialist disability service  Support within mainstream school  Specialist school

Respite care  NDIS-funded services  Community or family support

Other………………………………………………………………………………….

**Your child’s living arrangements**

At home  In Respite care  Out of home care  Group home  Full-time residential care

Other…………………………………………………….

Thank you for completing the survey. Your time and feedback are valued.

**If you would like to participate further**

You can contribute further by taking part in an ***in-depth phone interview***. This interview can be arranged separately and at your convenience. It would require 20-30 minutes of your time. Participation in an interview is entirely voluntary. It will be done confidentially and respect your privacy. It will not affect your child’s care.

Your input and contribution to this research project are greatly valued.

**I would like to participate further** ⎕ Yes ⎕No

**I can be contacted via**

Phone:

Email:

Other: *(please provide your preferred method of contact)*

If you would like to participate in this study, please complete the attached consent and send it back to us. You be will be contacted shortly.

**The researchers**

| Dr []  Advanced Paediatric Fellow | Dr []  Staff Specialist Anaesthetist  Dept of Anaesthesia | Dr []  Staff Specialist Paediatrician |
| --- | --- | --- |

|  |
| --- |
